# Editorial: Reviews in cancer metabolism

**DOI:** 10.3389/fonc.2023.1167484

**Published:** 2023-03-28

**Authors:** Nadia Jacobo-Herrera, Bela Ozsvari

**Affiliations:** ^1^ Unidad de Bioquímica, Instituto Nacional de Ciencias Médicas y Nutrición Salvador Zubirán, Mexico City, Mexico; ^2^ Translational Medicine, School of Science, Engineering and the Environment (SEE), University of Salford, Greater Manchester, United Kingdom

**Keywords:** cancer metabolism, metabolic therapy, metabolic reprogramming, resistance mechanisms, mitochondria

The Research Topic *Reviews in Cancer Metabolism* aimed to compile publications about the current state of the art on tumor cell metabolism, the implications on the aberrant functioning, tumor microenvironment, genomic features, and its impact on developing therapeutic targets.

The issue is enriched by seven articles from diverse research groups with remarkable contributions to the metabolism field in cancer, leaving open queries for discussion and future follow-up. The review suggests fresh ideas worth to be taken as cancer treatment targets.

According to the World Health Organization (1), cancer is the second leading cause of death from noncommunicable diseases worldwide, accounting for 9.3 million annually (1 https://www.who.int/data/gho/data/themes/topics/topic-details/GHO/ncd-mortality). Regardless of the daily work of the scientific community in tight collaboration with clinicians, deaths have not ceased (2). Understanding its origin, evolution, signaling pathways, and dysregulated metabolism are keys to preventing, controlling, or curing this atypic cell metabolism state, finding new therapeutic targets, and proposing new medications.

## 1 Cancer cell metabolism

Cancer is defined as a group of alterations in the functioning of a cell, i.e., its specialized metabolism allows it to have a high rate of proliferation under conditions not favorable for a normal cell. In general terms, cancer cell distinguishes by unlimited growth due to its ability to survive under stress conditions such as hypoxia, to evade cell death mechanisms, and its capacity of detaching from its place of origin to migrate and invade other tissues or organs, colonizing new sites and generating metastasis (3). Cell death evasion mechanisms and the high proliferative rate of tumor cells are due to their aberrant metabolism, which confers their success in propagation and metastasis. The incorrect functioning of cell metabolism leads to the activation or silence of several proteins and genes dysregulating essential pathways for the biosynthesis of macromolecules. Such is the case of cholesterol metabolism, in particular, the squalene monooxygenase enzyme (SQLE) reviewed by Zou et al. SQLE is an enzyme that participates in cholesterol synthesis and catalyzes the oxidation of squalene to (S)-2,3-epoxysqualene (4). When SQLE is dysregulated leads to cholesterol metabolism disorders associated with different diseases including cancer (5). The authors analyzed the main consequences of cancer derived from the wrong functioning of the enzyme SQLE. Clinically, the aberrant functioning of SQLE is related to poor prognosis and resistance to conventional treatments. Several preclinical studies with inhibitors of SQLE indicate diminished tumor growth, induce apoptosis, and G0/G1 cell cycle arrest in different neoplasias. Together these positive outcomes shift it into a potential pharmaceutic target.

One of the most aggressive and deadliest brain cancers is the glioblastoma multiforme (GBM). The diagnosis and prognosis are possible by genetic and histopathological tools. However, it is frequently resistant to conventional therapies, including surgery, chemotherapy, or radiation (6). El Khayari et al. collaborated on this issue with an assessment regarding the genetic alterations in the metabolism of GBM and the possibilities for treatment acknowledged to date. As previously mentioned, cancer cells demand high levels of glucose as an energy source to obtain ATP, which leads to a higher concentration of lactic acid, a process known as the “Warburg effect” or aerobic glycolysis (7). Thus, the malignancy of GBM depends on genes associated with glycolysis as well as lipid metabolism, both involved in maintaining optimal conditions in the tumor microenvironment and adaptation to hypoxia for growth. This review, as part of the topic about metabolism in cancer, clearly explains the role of the bioenergetic metabolism of the cancer cell as a target for new drugs, specifically dysregulated lipid synthesis and glycolysis, together with genomic aberrations.

## 2 Lipid metabolism in cancer cell

In the lipid metabolism scenario, occurs the adipose triglyceride lipase (ATGL) enzyme, which is regulated under hypoxia conditions. Zhang et al. revisited the state of the art of the ATGL role in tumorigenesis, cell proliferation, and metastasis in different cancers. ATGL regulates tumor metabolism under hypoxia conditions by the HIF-1/HIG-2/ATGL axis pathway, crucial for tumor cells to adapt to precarity oxygen levels. However, ATGL is controversial in cancer, it has a double activity, either tumor-promoting or tumor-suppressor depending on the cancer type. As illustrated in this paper, a pro-inflammatory environment and the presence of large amounts of lipids depend on tumor growth and even metastasis. An example is breast cancer, which complies with these two main requests. In summary, overexpression of ATGL leads to enhanced fatty acid oxidation that in consequence increases the metastatic ability of cancer cells.

## 3 Association mechanisms between cancer and diabetes

Recent studies have demonstrated the association of hyperglycemia and diabetes with poor prognosis in intraocular malignancies. Therefore, treatments targeting diabetes-related metabolic alterations, such as abnormal glucose metabolism, insulin resistance, and the IGF-1/IGF-1R signaling axis may have potential therapeutic benefits (8).

Gu et al. summarized in their review the common mechanisms shared by diabetes and intraocular malignancies, where they list all the potential drugs/inhibitors targeting the IGF-1/IGF-1R signaling axis, recently examined in either *in vitro* or in clinical studies. They also mention inhibitors of various glycolytic enzymes, GLUT1 inhibitors, and the use of metformin as possible therapeutics in intraocular cancers.

## 4 Targeting oxidative phosphorylation in ovarian cancer

Metabolic reprogramming and abnormal mitochondrial function of cancer cells have become novel targets in cancer metabolism (9). In their review, Wu et al. detail the phenomenon that certain chemotherapeutics can induce the selection of a drug-resistant subpopulation of cancer stem cells showing a metabolic shift to OXPHOS. These cells then stay dormant and can become the source of metastasis. Therefore, OXPHOS inhibition shows significant therapeutic potential in the prevention of the spread of cancer cells and the formation of secondary tumors.

## 5 Similarities between placenta and cancer that can aid cancer treatment

Embryogenesis and cancer metastasis share many cellular similarities. Pang et al. describe different aspects of these similarities in their review. Firstly, considering the placenta as a “pseudo-tumor” may help to understand why the accumulation of gene mutations in cancer becomes uncontrolled. Also, the decidua has the power of regulating placenta development. If further research reveals similarities between decidua and tumor microenvironment, it might be possible to reshape the tumor microenvironment and find novel ways for cancer treatments. Finally, changes in metabolites in the circulation of cancer cachexia patients and pregnant women involve many of the same molecules, such as hormones and exosomes.

## 6 Metabolic cancer therapy on tumor microenvironment

The tumor microenvironment is getting an increased attention in cancer therapies. Although, current knowledge of how the tumor microenvironment responds to cancer treatments is often limited. Emerging data suggest that metabolic treatments could substantially impact numerous non-cancerous cell types, including immune cells and fibroblasts both to enhance or reduce the efficacy of the intended therapeutic interventions (10). Therefore, understanding the metabolic vulnerabilities of both cancer and stromal cells can guide new treatment concepts and help better understand treatment resistance. Hyrossova et al. in their review detail the effects of various metabolic treatment strategies on the tumor microenvironment, highlighting inhibitors of glycolysis, OXPHOS, glutaminolysis, fatty acid, and nucleotide metabolism. In the figures, they distinctly presented compounds being tested either in preclinical or clinical trials. The review highlights metformin as the most studied OXPHOS inhibitor, and its effects on the TME are also the best characterized.

In summary, cancer metabolism has been recognized as one of the major mechanisms of resistance to current therapies. Also, a growing number of studies have shown that the metabolic phenotype of tumor cells is heterogeneous and distinct from normal tissue. Thus, targeting the metabolic differences between tumor and normal cells holds promise as a novel anticancer strategy. The reviews published in this article collection recapitulate the latest findings regarding these novel targets in cancer metabolism.

## Author contributions

NJ-H and BO contributed equally to the conception and writing of the manuscript. All authors contributed to the article and approved the submitted version.
